# Burnout Subtypes: Psychological Characteristics, Standardized Diagnoses and Symptoms Course to Identify Aftercare Needs

**DOI:** 10.32872/cpe.3819

**Published:** 2021-09-30

**Authors:** Gianandrea Pallich, Martin grosse Holtforth, Barbara Hochstrasser

**Affiliations:** 1 Center for Psychiatry and Psychotherapy, Private Hospital Meiringen, Meiringen, Switzerland; 2 Department of Clinical Psychology and Psychotherapy, University of Zurich, Zurich, Switzerland; 3 Department of Clinical Psychology & Psychotherapy, University of Bern, Bern, Switzerland; 4 Psychosomatic Medicine, Department of Neurology, Inselspital, Bern University Hospital, University of Bern, Bern, Switzerland; University of Tübingen, Tübingen, Germany

**Keywords:** depression, burnout, aftercare needs, diagnoses, symptoms, cluster analysis, subtypes

## Abstract

**Background:**

To better understand individual differences between burnout inpatients and improve individually tailored treatments in a psychiatric hospital, cluster analysis based on a number of self-report measures was used to investigate psychosocial characteristics of 96 participants.

**Method:**

Group membership was analyzed regarding associations with standardized measures of psychiatric and personality disorders. Moreover, symptom levels of burnout, depression, and general mental health were used to characterize the groups and to observe differential trajectories at admission, discharge, and follow-up.

**Results:**

As in previous research, we identified four subtypes that differed in comorbidity, psychological characteristics and treatment outcome. This calls for tailored interventions for the more vulnerable patients.

**Conclusion:**

The replicated and enriched characterization of burnout inpatients can help to optimally meet the differential needs of burnout patients.

## Highlights

- Four burnout subtypes were found: Functional, Dysfunctional, Straightforward Pragmatist, and Unhappy Altruist.
- Psychosocial characteristics and symptom levels at admission, discharge, and follow-up were described to better characterize the subtypes.
- The replicated and enriched characterization of burnout inpatients improves individually tailored treatments.

## The Importance of Burnout

The term burnout was introduced to the scientific discussion of psychological ailments in the 1970s by Freudenberger as a label of a negative affective state after having been exposed to continued work-related stress experiences (Freudenberger, 1974). Later, Maslach and colleagues (Maslach & Jackson, 1981) embossed the concept of burnout, recognizing emotional exhaustion, depersonalization, and a reduced sense of personal accomplishment with a sense of a diminished level of performance to be the key dimensions of this phenomenon. Criticisms of this definition notwithstanding, the related questionnaire, the Maslach Burnout Inventory (MBI), has become the gold standard in research and literature (Burisch, 2014). Since then, the phenomenon of burnout has been described in more than 60 different professions and professional subgroups (Kaschka, Korczak, & Broich, 2011), showing a prevalence of burnout varying between 3.5% and 50% (Nil et al., 2010). Not surprisingly, the conception and improvement of the clinical treatment of burnout inpatients have also become an important research focus (Hochstrasser, Von Bardeleben, Ruckstuhl, & Soyka, 2008).

## Long-Term Effects of an Inpatient Treatment Program for Burnout

Due to the heterogeneity and multifactorial etiology of burnout, a multimodal and individual treatment has been shown to be warranted (Hochstrasser et al., 2008; Schwarzkopf, Conrad, Straus, Porschke, & Von, 2016). Yet, the majority of studies on burnout interventions have not been performed with clinical samples, but in groups of volunteers who exhibited a level of burnout allowing them to maintain active engagement at work (Ahola et al., 2017; Awa et al., 2010; van der Klink et al., 2001). Patients with burnout who need inpatient care are those who are more afflicted, i.e., those who suffer from clinical burnout. Despite the importance of an adequate and effective inpatient treatment for burnout, to date, only few studies have examined the short- or long-term effects of inpatient treatment programs for burnout (Elkuch et al., 2010; Perski et al., 2017; Schwarzkopf et al., 2016). A previous study (Elkuch et al., 2010) examining a multimodal inpatient treatment at a private psychiatric hospital has found evidence of positive effects. Treatment included cognitive-behavioral individual and group psychotherapy, various relaxation techniques, body therapy, physical exercise, and psychopharmacological treatment. However, one limitation of the previous study was that assessments were performed only at admission and at follow-up, but not at discharge. Moreover, it has to be considered that the potential long-term effects of the inpatient treatment program and its sustainability may develop in the period between discharge and follow-up. Thus, assessing patients at admission, discharge, and at follow-up allows the examination of the short-term effects and the unfolding process of long-term effects more accurately. The expected results promise to yield valuable information serving the ongoing optimization of the future inpatient treatment of burnout.

## The Importance of Characterizing Patients Discharged From Inpatient Treatment for Burnout

Identifying burnout patients’ subtypes is crucial to tailoring treatment to patient characteristics and thereby improving burnout treatment. At an empirical level, some studies have identified subjects with burnout symptoms as one of several types of respondents in the workforce. Schaarschmidt and Fischer (2001) used self-report data on personal experiences with work-related stress and typical coping behaviors using the AVEM questionnaire (Work-related Behavior and Experience Patterns; German: Arbeitsbezogenes Verhaltens- und Erlebensmuster; Schaarschmidt & Fischer, 1996) to empirically categorize subjects in the workforce. The AVEM assesses stress experiences and coping behaviors in three domains and 11 subscales of six items each: *work commitment*, *resistance to stress*/*emotions*, and *subjective well-being* (Schaarschmidt & Fischer, 2001). The domains and subscales were identified by factor analyses of responses of 1598 subjects of diverse professions, and the AVEM has been subsequently used in various studies (Schulz et al., 2011; Voltmer et al., 2007, 2010, 2011). In the original study, Schaarschmidt and Fischer (2001) empirically identified four types of subjects based on scores in the 11 subscales: Healthy (Pattern G), Unambitious (Pattern S), Overexertion (Risk pattern A), and Burnout (Risk pattern B). In a recent study based on a sample of 1766 health care employees, Leiter and Maslach (2016) proposed five empirical profiles emerging from latent profile analyses of their dimensions of burnout (i.e., emotional exhaustion, depersonalization, and a reduced sense of personal accomplishment): Burnout (high on all three dimensions), Engagement (low on all three), Overextended (high on exhaustion only), Disengaged (high on cynicism only), and Ineffective (high on inefficacy only).

At a theoretical level, Montero-Marin and colleagues (Montero-Marín et al., 2009) proposed a three-partite classification of burnout patients based on a general proposal by Farber (1991): frenetic (involved and ambitious subjects who sacrifice their health and personal lives for their jobs); under-challenged (indifferent and bored workers who fail to find personal development in their job); and worn-out (subjects who feel they have little control over results and that their efforts go unacknowledged). Haberthür and colleagues (Haberthür et al., 2009) empirically classified burnout inpatients using self-report data on various interpersonal and intrapersonal aspects of functioning, such as social support, interpersonal problems, coping styles, emotion regulation, and motivational incongruence. The authors identified four groups by cluster analyses: Functional, Dysfunctional, Straightforward Pragmatist, and Unhappy Altruist.

For the current study, data were collected in the same private hospital and the same treatment unit as in the Haberthür et al. study. To our knowledge, the results of Haberthür et al.’s study (Haberthür et al., 2009) have not been replicated yet. The study did not assess standardized clinical diagnoses of psychiatric disorders and personality disorders, or comorbid somatic diagnoses, nor did it assess outcome at discharge.

The present study attempts to overcome these limitations and to replicate the former empirical classification of burnout inpatients to allow practitioners to tailor individual treatments to improve treatment outcomes. The self-reported person characteristics examined in Haberthür et al.’s study were motivational incongruence (motive satisfaction), interpersonal problems, social support, regulation of emotions, and coping styles. In the current study, the self-report measures used for clustering were the same as those used by Haberthür and colleagues, with the addition of as a self-report screening tool for personality dysfunction. Refining the clinical assessment methodology, structured interviews for psychiatric diagnosis and personality disorder were conducted. At admission, discharge, and follow-up, we assessed levels of depression, general symptoms and burnout.

## Aims

The aims of this study are: 1. To constructively replicate and improve a previously empirically derived description and categorization of burnout inpatients in an analogous treatment setting according to psychosocial parameters; 2. To characterize the patients and patient groups according to psychiatric diagnostic criteria; 3. To observe how group membership corresponds to different levels of psychological symptoms (depression and burnout) and general mental health at admission, discharge, and follow-up.

## Material and Method

### Sample, Treatment, and Recruitment

The present study was approved by the ethics committee of the canton Bern (Switzerland) and was conducted in the Private Hospital Meiringen. The sample comprised 96 inpatients of a specialized burnout ward. The therapeutic program includes individual psychotherapy, group therapy, relaxation techniques, body therapy, massages, sports activities and fitness instructions, psychopharmacotherapy, and selected interventions from complementary medicine (e.g., traditional Chinese medicine). A detailed description of the treatment program can be found in Hochstrasser et al. (2008).

The specialized burnout ward admits only patients being referred by a physician, having a burnout syndrome that arose primarily in the context of the work environment, and with a diagnostically confirmed burnout syndrome at admission evaluated in a clinical interview before admission. In this context, it is important to note that in the ICD-10, burnout is not considered to qualify as an independent psychiatric disorder but is listed as a syndrome being associated with difficulties pertaining to life circumstances (i.e., ICD-10, Z.73.0). An association of burnout with mental disorders, especially depression, has often been described, such that a recent overview on the overlap between depression and burnout postulated that clinical burnout corresponds to an atypical depression (Bianchi et al., 2015). Consequently, various comorbid primary psychiatric diagnoses according to ICD-10, Chapter F, were given on the basis of a clinical interview and in accordance with the patients’ symptomatic presentation at admission. To be included, patients had to be at least 18 years old. Patients were excluded if they exhibited current alcohol or drug addictions (if not stopped at admission), inability to participate in the treatment (e.g., due to psychological disorders or dementia), insufficient knowledge of the German language, or acute suicidality or psychotic symptoms. Between February 2017 and December 2017, a total of 173 inpatients were asked to participate in the study, a total of 113 inpatients gave their consent, and, due to missing data in cluster-relevant questionnaires, a total of 96 individuals, *n* = 96, *f* = 33 (34.4%), *m* = 63 (65.6%), were included in the analyses.

### Instruments

During the first week after admission, participants completed paper-pencil versions of different questionnaires and participated in two clinical interviews (Mini-DIPS and SCID-II) (Fydrich et al., 1997; Margraf, 2013) administered by the study psychologist. The discharge assessment was done in the last week of their stay, and the follow-up assessment was administered three months after discharge via paper-pencil questionnaires sent by mail with a pre-paid return envelope.

As in Haberthür et al. (2009), psychological characteristics were measured using the following self-report instruments: First, a short version of the Incongruence Questionnaire (German: Inkongruenzfragebogen, K-INK; grosse Holtforth & Grawe, 2003) was used to assess the degree of insufficient motivational satisfaction (approach incongruence and avoidance incongruence). The German 32-item short version of the Inventory for Interpersonal Problems was used to assess problematic interpersonal behaviors (IIP-SC; Soldz, Budman, Demby, & Merry, 1995; German: grosse Holtforth, 2005). The 32 items are an equivalent subset of the German IIP (IIP-D; Horowitz, Strauss, & Kordy, 2000). To measure the subjective appraisal of received or anticipated social support from persons in the social environment, the German short version of the Questionnaire of Social Support was used (German: Fragebogen zur sozialen Unterstützung, F-SOZU-K-22; Fydrich, Sommer, & Brähler, 2007). To evaluate different ways of coping with stressful situations (task-oriented, emotion-oriented, avoidance-oriented), the German version of the Coping Inventory for Stressful Situations (CISS; Kälin, 1995) was used. The Questionnaire for the Self-Evaluation of Emotional Competency (German: Fragebogen zur Selbsteinschätzung emotionaler Kompetenzen, SEK; Berking & Znoj, 2008) was used to measure deficits and resources in emotion regulation with the following scales: attention, awareness of bodily sensations, clarity, understanding, regulation, acceptance, resilience, self-support, and goal-oriented readiness to confront. The scale scores can be summarized by a total score. As mentioned before, previous studies did not assess personality and personality dysfunctions. To fill this gap, we added the Inventory of Personality Organization (IPO-16; Zimmermann et al., 2013) for clustering purposes. The 16-item short version of the Inventory of Personality Organization (IPO-16) is a self-report measure assessing the severity of personality dysfunction.

The level of symptoms and problems were assessed using the Beck Depression Inventory (BDI; Hautzinger et al., 1995), a brief version of the Symptom Checklist SCL-90 (SCL-9; Klaghofer & Brähler, 2001) and the Maslach Burnout Inventory – Human Services Survey (MBI-HSS; Maslach et al., 1997). The BDI is a self-report instrument assessing the degree of depressive symptomatology. The brief version of the Symptom Checklist assesses the general level of symptoms in one scale (Hautzinger et al., 1995). The MBI-HSS is considered the gold standard for burnout assessment and measures burnout in three dimensions (emotional exhaustion, depersonalization, and a sense of reduced personal effectiveness) (Maslach et al., 1997).

### Data Analytical Approach / Statistical Analysis

All statistical analyses were performed using the SPSS program (version 23.0) and Jamovi (version 0.8.6.0) (an interface program based on R). In a first step, a hierarchical cluster analysis (Ward’s Method) was performed to determine the appropriate number of clusters. Euclidean distance, which does not weigh outliers as strongly as the quadrated Euclidean distance, was used. According to these criteria, a cluster solution of four groups was considered optimal.

The following questionnaires and scales were used for clustering: the Incongruence Questionnaire (K-INK; Approach and Avoidance incongruence), the Inventory for Interpersonal Problems (IIP-SC/IIP-D; Dominance and Affiliation Dimensions), the Questionnaire of Social Support (F-SOZU-K-22; general score), the Self-Report Measure for the Assessment of Emotion Regulation Skills (SEK; general score); the Coping Inventory for Stressful Situations (CISS; task-oriented coping, emotion-oriented coping, avoidance-oriented coping) and the Inventory of Personality Organization (IPO-16; general score).

**Figure 1:**
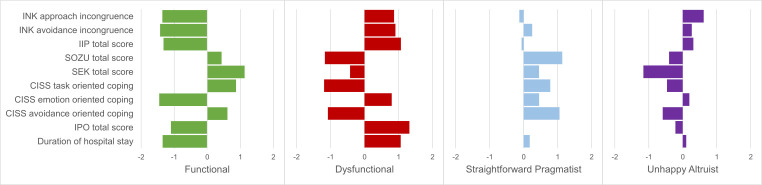
*z*-Standardized Levels of Psychological Characteristics Used for the Formation of the Four Groups and Duration of Hospital Stay (No Grouping Characteristic)

The number of clusters, i.e., four, corresponds to the number of clusters proposed by Elkuch et al. (2010). On the basis of the solutions suggested by the hierarchical cluster analysis, we further calculated confirmatory *k*-means cluster analyses for four cluster solutions. To exclude bias resulting from differing scaling of the various variables, all cluster analyses were performed using *z*-standardized values. Consequently, the values of the resulting groups were *z*-standardized and are presented as norm-related *z*-standardized values (see Figure 1). Descriptive statistics were used to describe the frequencies of different clinical diagnoses and personality disorder diagnoses (see Table 1).

**Figure 2:**
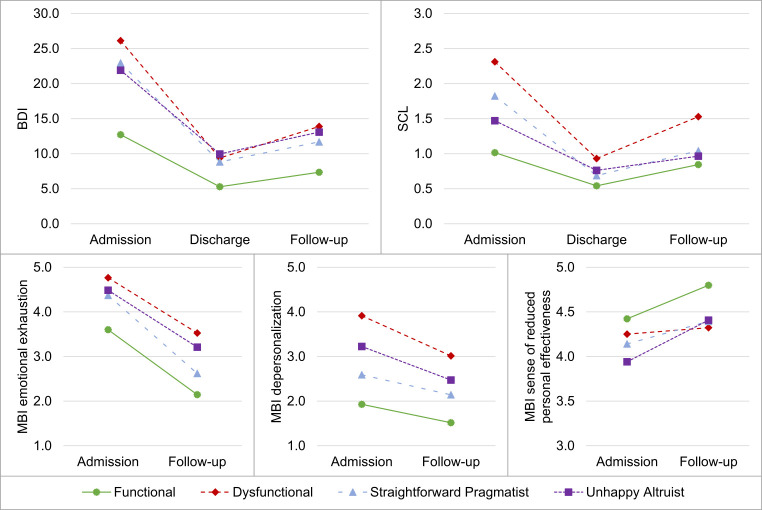
Repeated ANOVAs for the Four Groups (Functionals, Dysfunctionals, Straightforward Pragmatists and Unhappy Altruists) at Intake, Discharge and Follow-Up for BDI and SCL and at Intake and Follow-Up for the Three Dimensions of MBI (Emotional Exhaustion, Depersonalization and Sense of Reduced Personal Effectiveness)

BDI, SCL-9, and MBI-HSS were used to further characterize the groups (but not as factors to identify the groups) and to observe longitudinal development of symptoms. To evaluate differences in symptom levels among the resulting groups, we calculated a repeated measure analysis of variance (ANOVA) (see Figure 2).

**Table 1:** Main Psychiatric Diagnoses, Presence of an Additional Psychiatric Diagnosis, Presence of an Additional Somatic Diagnosis, and Personality Disorders for the Four Groups at Admission

	Total sample	Functionals	Dysfunctionals	Straightforward Pragmatists	Unhappy Altruists
Main Psychiatric Diagnoses (Mini-DIPS)					
F31.x (Bipolar disorder)	5.21% (*n* = 5)	5.88% (*n* = 1)	15.79% (*n* = 3)	0.00% (*n* = 0)	2.70% (*n* = 1)
F32.x (Major depressive disorder, single episode)	34.37% (*n* = 33)	17.65% (*n* = 3)	15.79% (*n* = 3)	43.48% (*n* = 10)	45.95% (*n* = 17)
F33.x (Major depressive disorder, recurrent)	46.87% (*n* = 45)	47.05% (*n* = 8)	57.90% (*n* = 11)	39.13% (*n* = 9)	45.95% (*n* = 17)
F43.x (Reaction to severe stress, and adjustment disorders)	6.25% (*n* = 6)	11.76% (*n* = 2)	5.26% (*n* = 1)	13.04% (*n* = 3)	0.00% (*n* = 0)
Missing	7.29% (*n* = 7)	17.65% (*n* = 3)	5.26% (*n* = 1)	4.35% (*n* = 1)	5.40% (*n* = 2)
**Presence of Comorbid Psychiatric Diagnoses (Mini-DIPS)**	33.33% (*n* = 32)	23.52% (*n* = 4)	42.08% (*n* = 8)	24.21% (*n* = 6)	37.80% (*n* = 14)
**Presence of Comorbid Somatic Diagnoses**	33.33% (*n* = 32)	35.28% (*n* = 6)	31.56% (*n* = 6)	21.75% (*n* = 5)	40.50% (*n* = 15)
**Avoidant personality Disorder (PD) & Obsessive-Compulsive PD (possibly comorbid with additional PD)**	6.25% (*n* = 6)	0.00% (*n* = 0)	21.05% (*n* = 4)	4.35% (*n* = 1)	1.70% (*n* = 1)
**Avoidant PD (possibly comorbid with additional PD)**	4.17% (*n* = 4)	5.88% (*n* = 1)	5.26% (*n* = 1)	0.00% (*n* = 0)	5.41% (*n* = 2)
**Obsessive-compulsive PD (possibly comorbid with an additional PD)**	23.96% (*n* = 23)	5.88% (*n* = 1)	21.05% (*n* = 4)	34.78% (*n* = 8)	27% (*n* = 10)
**Other PD**	5.21% (*n* = 5)	5.88% (*n* = 1)	10.52% (*n* = 2)	4.35% (*n* = 1)	1.70% (*n* = 1)
**No PD**	60.42 (*n* = 58)	82.35% (*n* = 14)	42.11% (*n* = 8)	56.52% (*n* = 13)	62.16% (*n* = 23)

## Results

### Sample Description

A total of 96 patients were included in the analyses. The mean age at admission was 48.02 years (*SD* = 8.78; 27.44 – 62.79 years). 33 (34.4%) of the participants were female, 63 (65.6%) were male. 50 married, 14 divorced, 25 singles, 3 separated, 1 widowed, and 1 unknown. The mean duration of the hospital stay was 57.31 days (*SD* = 16.04; 9 – 94 days). All the participants received medication during clinical stay. The duration between the time of discharge from the hospital and the follow-up assessment was 3 months. At follow-up, 14.6% participants were unemployed, 10.4% were fully employed, 33.3% were working part-time, 2.1% were working in their own household, 1.0% was in training for a different job, 3.1% were in a rehabilitation program, 6.3% were receiving a pension (i.e. an amount of money paid regularly by a government or company to somebody who has retired from work) or a disability pension (i.e. a form of pension given to those people who are permanently or temporarily unable to work due to a disability), and the employment status of 29.1% was unknown. In comparison to before the impatient stay, 6.3% were unemployed, 49.0% were fully employed, 20.8% were working part-time, 1.0% was working in their own household, none was in training, in a rehabilitation program, or receiving a pension / disability pension, and the employment status of 22.9% participants was unknown.

### Psychosocial Characteristics

Generally, the group labels are intended to be maximally comprehensive summaries of the respective characteristics. With the current sample and measures, we found that the obtained clusters corresponded closely to the previous grouping by Haberthür et al. (2009), so that we decided to keep the previous labels: (a) Functional, (b) Dysfunctional, (c) Straightforward Pragmatist, and (d) Unhappy Altruist.

#### Functionals

Participants categorized in this group, *n* = 17, *f* = 3 (17.6%), *m* = 14 (82.4%), experienced little avoidance incongruence (*z* = -1.43) and approach incongruence (*z* = -1.36). The patients mentioned few interpersonal problems (*z* = -1.32) and having good social support (*z* = 0.43). In addition, they reported good emotional competences (*z* = 1.13). The Functionals group used many task-oriented (*z* = 0.87) but just a few emotion-oriented (*z* = -1.45) coping strategies. They reported having little personality dysfunctions (*z* = -1.10). In general, they had a shorter stay in the hospital (*z* = -1.35).

Most of the participant in the group of Functionals had an F33.x (i.e., major depressive disorder, recurrent) diagnosis (*n* = 8, 47.05%), three (17.65%) had an F32.x (i.e., major depressive disorder, single episode) diagnoses, two (11.76%) an F43.x (i.e., reaction to severe stress, and adjustment disorders diagnoses) and one (5.88%) an F31.x (i.e., bipolar disorder) diagnoses. A total of four (23.52%) had a secondary psychiatric diagnosis (e.g., F10.1, F40.2, F41.0, F42.2). Six participants (35.29%) of this group additionally had one or more somatic diagnoses (e.g., E78.0, G44.0, G44.2, H95.1, I10.90, I10.91, R05, R73.1, Z61, Z62, Z73). For three participants it was not possible to use the Mini-DIPS for assessing standardized diagnoses.

Most of the participants categorized in the group of the Functionals showed no personality disorder (82.35%, *n* = 14).

#### Dysfunctionals

Compared to the other three groups, participants categorized in the group of Dysfunctionals, *n* = 19, *f* = 7 (36.8%), *m* = 12 (63.2%), showed the highest average approach incongruence (*z* = 0.87) as well as avoidance incongruence (*z* = 0.91). They showed strong interpersonal problems (*z* = 1.07). Additionally, they reported least social support (*z* = -1.17), generally insufficient emotional competence (*z* = -0.43) and mainly emotional coping (*z* = 0.80) and little task-oriented (*z* = -1.19) and avoidance-oriented (*z* = -1.08) coping strategies. In addition, they reported many personality dysfunctions (*z* = 1.32). In general, they had a longer stay in the hospital (*z* = 1.06).

Most of the participants in this group had an F33.x diagnosis (*n* = 11, 57.90%), three (15.79%) had an F32.x diagnosis, three (15.79%) an F31.x diagnosis and one (5.26%) an F43.x diagnosis. A total of eight (42.12%) had a secondary psychiatric diagnosis (e.g., F13.2, F40.2, F41.0, F41.1, F42.1, F43.1, F50.5). Six participants (31.59%) of this group additionally had one or more somatic diagnoses (e.g., A49.8, E03.9, E14.91, E78.5, G40.9, G43.9, G47.0, G47.39, I10.9, J45.0, M54.4, N48.0). One participant was not diagnosed systematically with Mini-DIPS.

The Dysfunctionals showed the highest association with a combination of avoidant and obsessive-compulsive personality disorders (21.05%, *n* = 4), and a high percentage had an obsessive-compulsive personality disorder (21.05%, *n* = 4) or other personality disorders (10.52%, *n* = 2).

#### Straightforward Pragmatists

On average, this group, *n* = 23, *f* = 13 (56.5%), *m* = 10 (43.5%), showed more avoidance incongruence (*z* = 0.25) than approach incongruence (*z* = -0.13). Generally, they reported a low level of interpersonal problems (*z* = -0.06). This group reported having good social support (*z* = 1.14). They generally had good emotional competences (*z* = 0.45). They reported using emotional coping (*z* = 0.46) and task-oriented coping (*z* = 0.78) at similar levels. This group showed average personality dysfunction (*z* = 0.00). The hospital stay was a little higher than average (*z* = 0.18).

Most of the participants in this group had an F32.x diagnosis (*n* = 10, 43.48%), nine (39.13%) had an F33.x diagnosis, three (13.04%) an F31.x diagnosis and none (0.00%) an F41.x diagnosis. A total of six (26.09%) had a secondary psychiatric diagnosis (e.g., F40.0, F40.2, F41.0, F44.4, F50.3). Five participants (21.74%) of this group additionally had one or more somatic diagnoses (e.g., E66.99, G35.9, G43.9, G47.31, H93.1, I10.90, I49.8). For one participant it was not possible to use the Mini-DIPS for assessing standardized diagnoses.

The group of the Straightforward Pragmatists showed the highest prevalence of obsessive-compulsive personality disorder compared to the other groups.

#### Unhappy Altruists

The members of this group, *n* = 37, *f* = 10 (27.0%), *m* = 27 (73.0%), showed higher average approach incongruence (*z* = 0.62) than avoidance incongruence (*z* = 0.27). Overall, they tended to show above-average scores in interpersonal problems (*z* = 0.31). Additionally, they reported bad social support (*z* = -0.40). Furthermore, this group showed an emotional competence below the average (*z* = -1.16). The members of this group primarily used emotion-oriented coping strategies (*z* = 0.19) and few task-oriented coping strategies (*z* = -0.46). This group showed little personality dysfunction (*z* = - 0.22). The hospital stay was a little longer than average (*z* = 0.10).

Seventeen participants (45.95%) of this group had an F32.x diagnosis. Seventeen participants 45.95%) had an F33.x diagnosis, and one participant (2.70%) had an F31.x diagnosis. No participants were diagnosed with F43.x in this group. A total of fourteen patients in this group (37.83%) had a secondary psychiatric diagnosis (e.g., F10.1, F13.2, F40.1, F40.2, F41.0, F44.2, F61.0). Fifteen participants (40.54%) had one or more somatic diagnoses in addition (e.g., D17.3, E11.90, E78.0, G25.0, G25.81, G43.0, G43.9, G47.1, G47.31, H93.1, H93.3, I10.90, K91.1, M17.9, M19.91, M53.0). For two participants it was not possible to use the Mini-DIPS for assessing standardized diagnoses.

The group of the Unhappy Altruists, similar to the group of the Straightforward Pragmatists, showed a higher percentage of obsessive-compulsive personality disorder compared to the other groups.

### Symptom Course in Groups of Burnout Patients

Data were analyzed using repeated measures ANOVAs for depression, general symptoms, and burnout with a within-subjects factor (admission, discharge, follow-up) and a between-subject factor of subtypes (Functional, Dysfunctional, Straightforward Pragmatist, and Unhappy Altruist). For missing values, list-wise deletion of cases was applied. For the three MBI dimensions, there were just two measured time points (admission and follow-up). Only for the repeated measures ANOVA for depression, the Mauchly’s test indicated that the assumption of sphericity had been violated, therefore degrees of freedom were corrected using Greenhouse-Geisser estimates of sphericity (ε = 0.876).

For the interested reader we report mean, median, standard deviation and range for BDI, SCL and MBI for the four groups (i.e. Functional, Dysfunctional, Straightforward Pragmatist and Unhappy Altruist) in the Supplementary Material

#### Depression (Beck Depression Inventory, BDI)

A repeated measures ANOVA (see Figure 2) with a Greenhouse-Geisser correction showed that mean depression scores differed significantly between time points, *F*(1.75, 127.86) = 117.55, *p* < .001, $\eta_{p}^{2}$ = .617, and between groups, *F*(3, 73) = 4.46, *p* < .01, $\eta_{p}^{2}$ = .158. The interaction between time and groups was also significant, *F*(5.25, 127.86) = 2.51, *p* < .05, $\eta_{p}^{2}$ = .093. Post hoc tests revealed that the depression scores for the Functionals group was the lowest and differed highly significantly from those of all other groups regarding (*p* < .001). For the remaining groups, depressive symptoms were higher at admission, all at similar and non-significantly different levels. At discharge, levels of depressive symptoms did not differ significantly between the four groups. However, at follow-up, the average depression levels of the Functional and Dysfunctional groups differed significantly (*p* < .05), and also a significant difference between Functionals and Unhappy Altruists (*p* < .05) was found. All patient groups showed a significant decrease of depressive symptoms from admission to discharge (*p* < .05). Whereas Functionals, Straightforward Pragmatists, and Unhappy Altruists reported no significant increase of depressive symptoms between discharge and follow-up, the Dysfunctionals showed a significant increase of depressive symptoms (*p* < .05).

#### General Symptoms (Brief Symptom Checklist, SCL)

A repeated measures ANOVA (see Figure 2) showed that the mean general symptoms scores differed significantly between time points, *F*(2, 154) = 53.23, *p* < .001, $\eta_{p}^{2}$ = .409, and between groups, *F*(3, 77) = 7.24, *p* < .001, $\eta_{p}^{2}$ = .220. The interaction between time and groups was also significant, *F*(6, 154) = 2.60, *p* < .05, $\eta_{p}^{2}$ = .092. The Functionals had the lowest symptom level, and the Dysfunctionals the highest at admission, discharge, and follow-up. Straightforward Pragmatists and Unhappy Altruists showed similar levels of general symptoms, with levels being lower than those of Dysfunctionals but higher compared to the Functional group at all three measurement points. Post hoc tests revealed that at admission, the Functionals differed highly significantly regarding the general symptoms from the Dysfunctional and Straightforward Pragmatists (*p* < .01) and significantly from the Unhappy Altruists (*p* < .05). The group of Dysfunctionals differed highly significantly from the group of Unhappy Altruists (*p* < .01) and significantly from the group of Straightforward Pragmatists (*p* < .05). Regarding general symptoms at discharge, no significant differences could be found between the four groups. At follow-up Dysfunctionals differed from all other groups (*p* < .05). All groups showed a significant decrease of symptoms between admission and discharge (*p* < .05). Whereas Dysfunctionals and Straightforward Pragmatists showed significant increases of symptoms between discharge and follow-up (*p* < .05), this was not the case for the Functionals and the Unhappy Altruists.

#### Burnout (Maslach Burnout Inventory, MBI)

A repeated measures ANOVA for the dimension of emotional exhaustion showed a significant difference between admission and follow-up, *F*(1, 71) = 56.87, *p* < .001, $\eta_{p}^{2}$ = .445, and between groups, *F*(3, 71) = 3.10, *p* < .05, $\eta_{p}^{2}$ = .116. The interaction between time and groups was not significant. Post hoc tests revealed that regarding emotional exhaustion, all groups showed a significant (*p* < .01) decrease between admission and follow-up. Functionals and Dysfunctionals showed a significantly different level of emotional exhaustion at admission (*p* < .01).

A second repeated measures ANOVA for MBI for the dimension of depersonalization showed a significant difference between admission and follow-up, *F*(1, 71) = 11.83, *p* < .001, $\eta_{p}^{2}$ = .143, and between groups, *F*(3, 71) = 4.54, *p* < .01, $\eta_{p}^{2}$ = .161. The interaction between time and groups was not significant. Post hoc tests showed that Functionals differed from Dysfunctionals and Unhappy Altruists significantly concerning depersonalization at admission (*p* < .05). Additionally, Dysfunctionals differend from Straightforward Pragmatists (*p* < .05). Dysfunctionals’ and Unhappy Altruists’ level of depersonalization decreased significantly (*p* < .05) between admission and follow-up.

A last repeated measures ANOVA for the third MBI dimension, the sense of reduced personal effectiveness, showed a significant difference between admission and follow-up, *F*(1, 71) = 4.61, *p* < .05, $\eta_{p}^{2}$ = .061, and no significant difference between groups. The interaction between time and groups was not significant. In a post hoc analysis, Unhappy Altruists reported, as the only group, a significant (*p* < .05) improvement in the sense of reduced personal effectiveness.

## Discussion

In the present study, we set out to reproduce the results and improve the previous descriptions of burnout inpatients of Haberthür and colleagues (2009). First, a cluster analysis was used to group burnout patients. Second, we characterized the burnout patients according to psychosocial parameters. Additionally, the groups were described regarding their residual symptoms at admission, discharge, and follow-up. Finally, we described the psychiatric, somatic, and personality disorder diagnoses of the sample. Four groups were identified based on clustering (i.e., Functional, Dysfunctional, Straightforward Pragmatist, and Unhappy Altruist).

The Functional group was characterized by low levels of motivational incongruence, interpersonal problems, emotion-oriented coping, and personality dysfunction and showed good social support, emotional regulation, and mainly task-oriented and avoidance-oriented coping. The members of the Dysfunctional group had an almost reversed profile showing high levels for incongruence, interpersonal problems, emotion-oriented coping, and personality dysfunction in addition to low social support, emotional regulation, task- and avoidance-oriented coping. The other two groups (Straightforward Pragmatists and Unhappy Altruists) did not show characteristically extreme values in the above-mentioned variables. Straightforward Pragmatists reported good social support, emotional competences, and using all three coping strategies. Unhappy Altruists reported levels of incongruence and interpersonal problems a little above average, low social support, and emotional competences as well as stronger use of emotion-oriented than task-oriented or avoidance-oriented coping strategies.

All psychosocial characteristics of the Functional group could be reproduced without exception as described by Haberthür and colleagues (2009). The group of the Dysfunctionals had similar psychosocial parameters as found in the previous study, with the exception of task-oriented coping that was found to be low instead of average. For the other groups (i.e., Straightforward Pragmatists and Unhappy Altruists), we found similar psychosocial parameters as in the previous study. Only the emotional competence of the Straightforward Pragmatists was found to be high and not average, and reported levels of emotional competence and social support of the Unhappy Altruists were found to be low instead of average.

Above and beyond replicating the description by psychosocial parameters, also psychiatric, somatic, and personality disorder diagnoses were assessed for the four subtypes of burnout patients. The standardized assessment of psychiatric diagnoses showed most of the participants of the groups of Functionals and Dysfunctionals having a recurrent major depressive disorder. Furthermore, most of the participants in the group of the Straightforward Pragmatists had a single major depressive disorder. Finally, the participants of the group of the Unhappy Altruists had the same frequency of recurrent major depressive disorder and single major depressive disorder. Interestingly, all groups showed some comorbidity of somatic diagnoses. Around one third of the Functionals and Dysfunctionals had, in addition to psychiatric diagnoses, also a somatic diagnosis, whereas around one quarter of the group of the Straightforward Pragmatists had a somatic diagnosis. Finally, 40.50% of the Unhappy Altruists had a diagnosis of a somatic disorder.

In previous studies, a high level of overlap between burnout and depression symptomatology was found in all groups of burnout patients, to the point that it has been suggested that clinical burnout may rather be a form of depression (Bianchi et al., 2015). The DGPPN (Berger et al., 2012) proposed to consider depression as a common consequence of prolonged burnout. Yet, the temporal relationship between burnout and depression remains unclear (Ahola & Hakanen, 2007). In order to require inpatient care, burnout patients are likely to be more strongly affected and more impaired regarding daily functioning, which might bias the sample we examined towards those with a depressive disorder or other mental disorders.

Functionals showed almost no personality disorders, Dysfunctionals had a higher prevalence of personality disorders (especially Avoidant PD in combination with Obsessive-compulsive PD and Obsessive-compulsive PD), and both Straightforward Pragmatists and Unhappy Altruists showed a high prevalence of Obsessive-compulsive PD. The results of the IPO-16, assessing the severity of personality dysfunction, seem to confirm these findings, indicating similar personality dysfunctions as found through SCID-II for the four groups (very low for Functionals, very high for Dysfunctionals, average for Straightforward Pragmatists, and low for Unhappy Altruists). These findings could be relevant for planning tailored treatments for burnout patients considering that treatment of personality disorder is a major goal of psychotherapy interventions for all groups except the Functionals.

Symptom level at admission, discharge, and follow-up was assessed for the four groups using the BDI, the SCL, and the MBI. Generally, all four groups improved significantly between admission and discharge regarding depressive symptoms and overall symptom level. Dysfunctionals showed an increase of depressive symptoms between discharge and follow-up. Dysfunctionals and Straightforward Pragmatists showed a significant increase of general symptoms between discharge and follow-up. This worsening should be considered for discharge planning for the group of Straightforward Pragmatists and even more so for the Dysfunctionals. Particularly for Dysfunctionals, increased attention to discharge planning and more intensive support after leaving the clinic seems indicated.

### Practical Implications

This study suggests that it is of great importance to attend to the relevant psychological characteristics of burnout patients, and that applying our categorization early in the process could improve the success of treatment and discharge planning. This may be done by clinical judgment or, if available, also by using structured assessment tools. Depending on burnout group membership, the needs of inpatients are likely to be different.

As indicated by group label, Functionals generally show more benign characteristics and are more likely to improve during the inpatient treatment, an effect that appears sustained during follow-up. Under the perspective of optimal resource allocation, frequent monitoring of patients’ mental health might suffice to meet their needs for care after discharge.

In contrast, our data suggest that the group of Dysfunctionals may need the most intensive inpatient treatment and well-organized psychosocial aftercare. Our data suggest that increasing the level of a patient’s motivational satisfaction needs to be an important treatment goal, and the identification of the individual sources of motivational incongruence will help to select targeted interventions. Also, due to strong interpersonal problems, lower levels of perceived social support, and insufficient emotional competence, assertiveness training (Rakos, 1991), activation of the patient’s social network (Perry & Pescosolido, 2015; Pescosolido & Levy, 2002; Smith & Christakis, 2008), as well as training of emotional skills (Berking, 2015; Cherniss, 2000; Pallich et al., 2020) might be suitable interventions. The enhancement of a task-oriented coping style and related skills may be an important target for longer-term treatment of this group.

Whereas Straightforward Pragmatists showed rather unproblematic profiles at admission with regard to psychological characteristics, they showed significant increases of symptoms between discharge and follow-up. For this reason, assessment during inpatient treatment should exceed patient self-reports to not miss relevant stressors that patients might not be able or willing to report. In addition, the formulation of crisis-response plans may be indicated, as well as close symptom monitoring after discharge.

Finally, also Unhappy Altruists, who show interpersonal problems above the average, bad social support, and emotional competence below the average, could profit from assertiveness training (Rakos, 1991) and a training of emotional competence (Berking, 2015; Cherniss, 2000; Pallich et al., 2020). This group, who showed higher levels of approach incongruence and avoidance incongruence, indicating dissatisfaction of motives, should be analyzed more deeply during treatment. Targeted interventions may be selected after recognizing individual sources of motivational incongruence to increase the level of patients` motivational satisfaction. Finally, especially for the group of Unhappy Altruists, who show the highest rate of comorbid somatic diagnoses, it is suggested to consider specific interventions and treatment for somatic problems.

Additionally, an assessment of personality disorders (Fydrich et al., 1997) seems indicated particularly for Straightforward Pragmatists and Unhappy Altruists, who generally show a high percentage of comorbidity with personality disorder diagnoses. During the inpatient stay and after discharge, a long-lasting psychotherapeutic treatment with a focus on personality disorder should be implemented (Sachse, 2013). Especially strategies for avoidant and obsessive-compulsive personality disorders could play an important role (Sachse, 2013).

The present findings should be considered in light of some methodological limitations. First, the sample was relatively small and recruited in only one clinic, and sample sizes of the groups resulting from cluster analysis differed considerably (*n* = 17 to *n* = 37). Second, because of the heuristic nature of this study, we omitted corrections of the significance levels for multiple testing (e.g., Bonferroni). Additionally, the intervals between assessments were different across the sample as the length of inpatient stay varied. This may have affected symptom scores at discharge and follow-up. Furthermore, follow-up data were collected only three months after discharge and the patient sample consists of patients of an inpatient ward of one psychiatric hospital in one country and is therefore not representative for the population of burnout patients.

Future research should replicate the classification of former patients in different, larger, and more diverse samples. An additional follow-up later in time could be more informative regarding relapse and promote the development of tailored interventions for the different groups. Following up inpatients months or even years after discharge could provide further information about potential difficulties patients may encounter in the long run regarding the course of symptoms and especially relapse risk for different groups. Additionally, different and tailored intervention programs for acute treatment and maintenance care for the different burnout types should be developed and tested. Those programs should focus on the individual needs and the tailored therapeutic interventions of the different groups, as mentioned above. We assume that observing such a differentiated treatment approach will increase the probability of an effective and long-lasting successful treatment outcome. Further long-term data collection will allow evaluating the effects of more tailored programs on the basis of assessed burnout subtypes in service of further optimizing the acute treatment and aftercare of burnout patients. The development of tailored treatment programs for the different subtypes of burnout patients and their long-term evaluation will be an important next step to optimize the acute treatment and aftercare of burnout patients.

### Conclusion

To the best of our knowledge, this is the first study to constructively replicate and improve the attempt to categorize burnout inpatients of Haberthür and colleagues (2009). Overall, we were able to replicate and improve the characterization of the four different groups: Functional, Dysfunctional, Straightforward Pragmatist, and Unhappy Altruist. Additionally, we described psychiatric, somatic, and personality disorder diagnoses. We further showed the symptoms course in the four groups of burnout patients. These findings support the proposition that burnout is a heterogeneous phenomenon. For clinicians it is necessary to consider these different characteristics of burnout inpatients in order to assure an individually tailored treatment program and corresponding discharge and aftercare planning. Future research should focus on tailored treatment programs depending on different subtypes of burnout patients.

## Supplementary Materials

In the Supplementary Materials we report mean, median, standard deviation and range for BDI, SCL and MBI for the four groups (i.e. Functional, Dysfunctional, Straightforward Pragmatist and Unhappy Altruist) (for access see Index of Supplementary Materials below).

10.23668/psycharchives.5093 Supplement 1 Supplementary materials to "Burnout subtypes: Psychological characteristics, standardized diagnoses and symptoms course to identify aftercare needs" [Additional results]

PallichG.
grosse HoltforthM.
HochstrasserB.
 (2021). Supplementary materials to "Burnout subtypes: Psychological characteristics, standardized diagnoses and symptoms course to identify aftercare needs"
[Additional results]. PsychOpen. 10.23668/psycharchives.5093
PMC966723236398100
