# Supplementary material for: Burnout Subtypes: Psychological Characteristics, Standardized Diagnoses and Symptoms Course to Identify Aftercare Needs
Source: Clin Psychol Eur. 2021 Sep 30;3(3):e3819. doi: 10.32872/cpe.3819 (PMC9667232; doi:10.32872/cpe.3819)
Supplement: Supplement 1 [file cpe-03-3819-s01.pdf]

Burnout Subtypes: Psychological Characteristics, Standardized Diagnoses and Symptoms Course to Identify Aftercare Needs

Gianandrea Pallich [1 2], Martin grosse Holtforth [3 4], Barbara Hochstrasser [1]

[1] Private Hospital Meiringen, Center for Psychiatry and Psychotherapy, Meiringen, Switzerland

[2] University of Zurich, Clinical Psychology and Psychotherapy, Zurich, Switzerland

[3] University of Bern, Department of Clinical Psychology & Psychotherapy, Bern, Switzerland

[4] Inselspital, Psychosomatic Competence Center, Bern, Switzerland

Manuscript published in:

Clinical Psychology in Europe

https://doi.org/10.32872/cpe.3819

Corresponding author:

Gianandrea Pallich, University of Zurich, Clinical Psychology and Psychotherapy, Zurich, Switzerland

E-Mail: g.pallich@psychologie.uzh.ch

Supplementary Material

Table 1: mean, median, standard deviation and range for BDI at admission, discharge and follow-up for the four groups (i.e. Functional, Dysfunctional, Straightforward Pragmatist and Unhappy Altruist).

		**BDI**		
	**Groups**	**Admission**	**Discharge**	**Follow-up**
Mean	Functionals	13.1	6.68	7.36
	Dysfunctionals	26.4	9.59	13.9
	Straightforward Pragmatists	22.5	9.76	11.6
	Unhappy Altruists	23.8	9.74	12.7
Median	Functionals	13.0	6.00	5.50
	Dysfunctionals	27.0	10.0	14.5
	Straightforward Pragmatists	21.5	9.00	10.0
	Unhappy Altruists	21.0	6.00	11.5
Standard deviation	Functionals	6.32	6.46	6.39
	Dysfunctionals	6.13	5.78	9.57
	Straightforward Pragmatists	7.57	7.67	7.99
	Unhappy Altruists	9.01	8.64	9.43
Range	Functionals	23.0	22.0	21.0
	Dysfunctionals	22.0	20.0	34.0
	Straightforward Pragmatists	31.0	29.0	34.0
	Unhappy Altruists	33.0	27.0	33.0

Table 2: mean, median, standard deviation and range for SCL at admission, discharge and follow-up for the four groups (i.e. Functional, Dysfunctional, Straightforward Pragmatist and Unhappy Altruist).

		**SCL**		
	**Groups**	**Admission**	**Discharge**	**Follow-up**
Mean	Functionals	1.09	0.579	0.844
	Dysfunctionals	2.30	0.922	1.53
	Straightforward Pragmatists	1.80	0.695	1.04
	Unhappy Altruists	1.59	0.772	1.01
Median	Functionals	1.00	0.444	0.778
	Dysfunctionals	2.22	0.778	1.17
	Straightforward Pragmatists	1.72	0.556	0.944
	Unhappy Altruists	1.56	0.556	0.667
Standard deviation	Functionals	0.413	0.507	0.546
	Dysfunctionals	0.673	0.550	1.11
	Straightforward Pragmatists	0.777	0.566	0.687
	Unhappy Altruists	0.717	0.573	0.747
Range	Functionals	1.33	1.44	1.56
	Dysfunctionals	2.89	1.56	3.33
	Straightforward Pragmatists	3.11	3.00	2.56
	Unhappy Altruists	2.44	2.11	2.22

Table 3: mean, median, standard deviation and range for the three dimensions of MBI (i.e. Physical Fatigue, Emotional Exhaustion, Cognitive Weariness) at admission and follow-up for the four groups (i.e. Functional, Dysfunctional, Straightforward Pragmatist and Unhappy Altruist).

		**MBI**					
		**Emotional Exhaustion**		**Depersonalization**		**Reduced Personal Effectiveness**	
	**Groups**	**Admission**	**Follow-up**	**Admission**	**Follow-up**	**Admission**	**Follow-up**
Mean	Functionals	3.66	2.14	2.02	1.51	4.49	4.80
	Dysfunctionals	4.79	3.52	3.94	3.01	4.29	4.32
	Straightforward Pragmatists	3.96	2.62	2.57	2.14	4.06	4.39
	Unhappy Altruists	4.54	3.11	3.35	2.40	3.91	4.43
Median	Functionals	3.60	1.70	1.60	1.40	5.00	5.25
	Dysfunctionals	5.20	3.90	4.40	2.70	4.30	4.50
	Straightforward Pragmatists	4.40	2.80	2.20	1.50	4.05	4.25
	Unhappy Altruists	4.80	3.10	3.40	1.70	4.00	4.92
Standard deviation	Functionals	1.38	1.51	1.45	1.37	1.15	1.37
	Dysfunctionals	1.24	1.83	1.57	1.78	1.03	1.38
	Straightforward Pragmatists	1.58	1.61	1.36	1.60	1.27	1.18
	Unhappy Altruists	1.04	1.86	1.40	1.81	1.43	1.38
Range	Functionals	4.60	4.80	5.40	4.20	4.30	4.17
	Dysfunctionals	4.60	5.60	5.20	6.00	4.30	3.83
	Straightforward Pragmatists	5.60	5.00	5.00	4.60	4.40	3.50
	Unhappy Altruists	3.80	6.00	5.40	5.80	4.90	4.17
